# Temporal antibody responses to SARS-CoV-2 in patients of coronavirus disease 2019

**DOI:** 10.1038/s41421-020-00209-2

**Published:** 2020-09-15

**Authors:** Min Huang, Qing-Bin Lu, Han Zhao, Yulan Zhang, Zhiwei Sui, Liqun Fang, Di Liu, Xiulian Sun, Ke Peng, Wei Liu, Wuxiang Guan

**Affiliations:** 1 Department of Laboratory Medicine, Tongji Hospital, Tongji Medical College, HUST, Wuhan, Hubei 430030 China; 2 Department of Laboratorial Science and Technology, School of Public Health, Peking University, Beijing 100871, China; 3 School of Mathematical Sciences, Beijing Normal University, Beijing 100875, China; 4 CAS Key Laboratory of Special Pathogens and State Key Laboratory of Virology, Wuhan Institute of Virology, Center for Biosafety Mega-Science, Chinese Academy of Sciences, Wuhan, Hubei 430071 China; 5 Center for Advanced Measurement Science, National Institute of Metrology, Beijing 100029, China; 6 State Key Laboratory of Pathogen and Biosecurity, Beijing Institute of Microbiology and Epidemiology, Beijing 100071, China

**Keywords:** Immunology, Autoimmunity

Dear Editor,

The coronavirus disease 2019 (COVID-19), caused by the severe acute respiratory syndrome coronavirus 2 (SARS-CoV-2), has become a pandemic within a few months. Up to 31st July 2020, it had affected over 17,000,000 individuals worldwide causing over 670,000 deaths^1^. In most cases, COVID-19 is associated with mild symptoms, while some patients develop severe disease^2,3^. Previous investigations of COVID-19 patients have demonstrated that SARS-CoV-2 IgM antibodies are usually detectable a week after illness onset and can persist for one month after infection^4^. IgG antibody can be detected 10 days from illness onset, which may last for a longer period^4^. It was also shown that antibody titers are higher and longer-lived in more severely ill patients than in mildly ill patients^5^, some of the latter do not develop a detectable antibody response^6^. However, data on the simultaneous evaluation of cellular immune response, cytokine production, and specific antibody were lacking^7^.

Here, we assessed the longitudinal clinical, laboratory, viral, and immunological data from 366 COVID patients. Severity of the COVID-19 patients was defined according to the “Diagnosis and Treatment Scheme of New Coronavirus Infected Pneumonia”^8^. An IgM and IgG antibody detection kit was employed to detect the antibody responses in COVID-19 patient serum. A total of 366 RT-PCR confirmed COVID-19 patients were enrolled. The median age was 62 years and 177 (48.4%) were male (Supplementary Table S1). The delay from symptom onset to the hospital admission was 20 days (interquartile range (IQR) 10–29). Among these, 65 patients had documented diabetes and 136 had hypertension. 144 patients were diagnosed as having severe disease and 222 having mild disease.

The anti-SARS-COV-2 IgM antibody profile was illustrated (Fig. 1a). Based on the calculation of every 10 days, both the IgM positive rates (82.1%) and the antibody level (geometric mean reciprocal titer (GMRT) 39.1, 95% confidence interval (CI) 8.1–190.6) peaked at 20–30 days and decreased thereafter with 50% tested positive and the GMRT evaluated to be 9.2 (95% CI: 2.2–39.6) at 60-day after symptom onset, which is the last observation point of the study. The anti-SARS-COV-2 IgG response profile was also calculated every 10 days (Fig. 1b). The positive rates peaked at 60–70 days (100%). Based on the GMRT estimation, the IgG antibody level peaked at 20–30 days of illness (GMRT 138.0, 95% CI 66.0–288.6) and decreased slowly thereafter (GMRT 94.3, 95% CI 61.6–145.5).

**Fig. 1:**
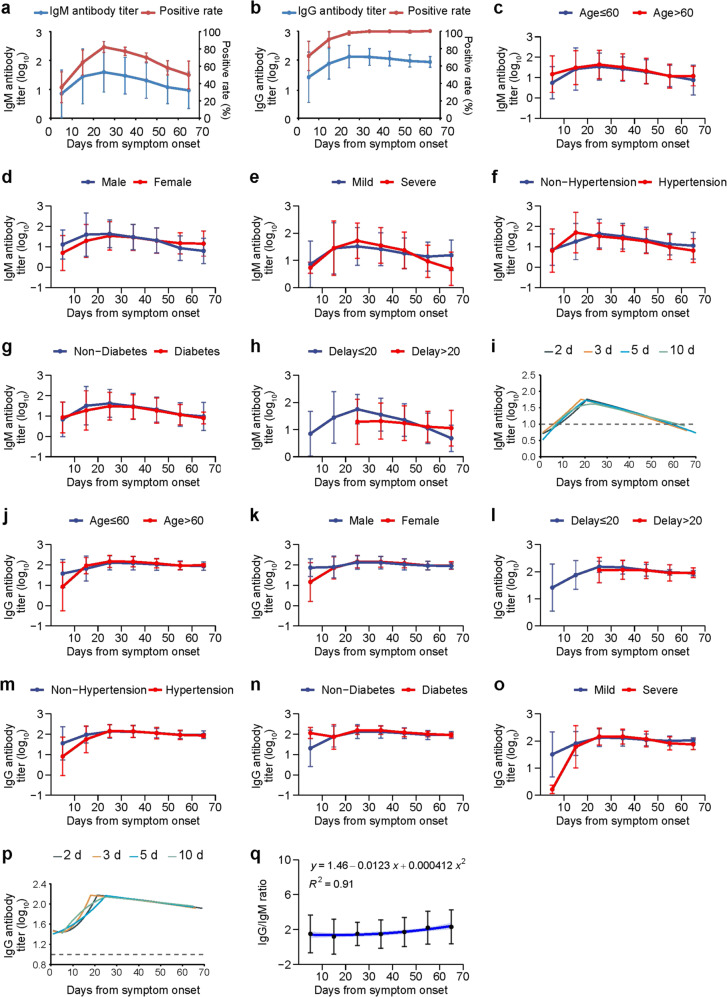
Dynamic profile on antibody responses to SARS-CoV-2 in COVID-19 patients. The IgM and IgG levels in the COVID-19 patient serum was determined with a chemiluminescence assay. A total of 366 RT-PCR confirmed patients were enrolled. **a** IgM antibody titer (blue) and positive rate (red); **b** IgG antibody titer (blue) and positive rate (red); **c** IgM level in patients with age ≤60-year or age >60-year old; **d** IgM level in 177 male and 189 female patients; **e** IgM level in patients with 222 mild and 144 severe symptoms; **f** IgM level in 136 patients with and 230 patients without hypertension; **g** IgM level in 65 patients with and 301 patients without diabetes; **h** IgM level in 212 patients with ≤20 days and 154 patients with >20 days delay from symptom onset to admission; **i** quadratic fitting curves of IgM titer calculated based on every two-day, three-day, five-day, and ten-day time points; **j** IgG level in patients with age ≤60-year or age >60-year old; **k** IgG level in the male or female patients; **l** IgG level in 212 patients with ≤20 days and 154 patients with >20 days delay from symptom onset to admission; **m** IgG level in 136 patients with and 230 patients without hypertension; **n** IgG level in 65 patients with or 301 patients without diabetes; **o** IgG level in patients with mild (222) or severe (144) symptoms; **p** quadratic fitting curve of IgG titer calculated based on every two-day, three-day, five-day, and ten-day time points; **q** IgG/IgM ratio was calculated as log_10_(1/IgG) divided by log_10_(1/IgM); the mean and standard deviation were presented for IgG/IgM ratio; the fitting curve for IgG/IgM ratio and 95% CI (light blue area) were plotted. The GMRT and standard deviation were presented for antibody titers. The positive rate and 95% CI were presented for IgM or IgG. The IgM and IgG antibody reciprocal titers were log-transformed to allow for comparisons of GMRT across groups by GEE (**c**–**h** and **j**–**n**). The quadratic fitting curves were performed for IgM (**i**) or IgG (**p**) antibody titers in the rising stage and falling stage. Calculation was performed based on every two-day, three-day, five-day, and ten-day points, of which the ten-day points results showed the highest R^2^ (0.99 for IgM and 0.93 for IgG) and was used for the analysis. The measurement was performed once with the coefficient of variation value of around 5%. CI confidence interval, GMRT geometric mean reciprocal titer, GEE generalized estimation equation.

The magnitude of IgM antibody was delineated in relation with five variables (age, gender, the delay from symptom onset to admission, comorbidities, and disease severity) (Fig. 1c–h and Supplementary Table S2). Their effects on the antibody responses were estimated by performing generalized estimating equation (GEE) analysis. Before the peaking point, the patients of younger age (Fig. 1c), female (Fig. 1d), and those with mild disease (Fig. 1e) produced lower IgM GMRT (all *P* < 0.05).

After the peak point, a lower GMRT of IgM antibody was also observed for younger age, and additionally for patients with hypertension or patients with longer days from symptom onset to admission (Fig. 1c, f, h and Supplementary Table S2) (*P* < 0.05, *P* < 0.001, and *P* < 0.05), respectively. By applying GEE analysis, significant effects on antibody level of IgM were determined from age, days from symptom onset to admission and disease severity (*P* < 0.05, *P* < 0.001, and *P* < 0.05), respectively. The maximum of IgM antibody titer for each patient was compared, and only the patients with >20 days from symptom onset to admission had a lower peaking level of IgM antibody titer (*P* < 0.05, Supplementary Table S3). The quadratic fitting curves were performed on titers calculated every 2-day, 3-day, 5-day, and 10-day points, respectively, with the curve based on 10-day point attaining the highest R^2^. Accordingly, the half-life of IgM was estimated as ~51 days and the diminish time was estimated to be around 62 days post symptom onset (Fig. 1i and Supplementary Table S4).

The IgG antibody response was similarly delineated by the days from disease onset to detection (Fig. 1j–o). Different from IgM antibody, a higher GMRT of IgG antibody was observed in patients with diabetes (*P* < 0.05) before the peak point (Fig. 1n), while significant higher GMRT of IgG was observed in patients with older age (*P* < 0.01, Fig. 1j), shorter days from symptom onset to admission (*P* < 0.001, Fig. 1l) or diabetes (*P* < 0.001, Fig. 1n) after the peak point. By applying GEE analysis, significant effects on GMRT of IgG were determined from age, diabetes, and disease severity (*P* < 0.01, *P* < 0.001, and *P* < 0.05). The maximum of IgG antibody titer for each patient was compared, and only patients with younger age and >20 days from symptom onset to admission had a lower peak level of IgG (*P* < 0.05 and *P* < 0.001, Supplementary Table S3). The quadratic fitting curves were performed on titers calculated every ten-day points, which showed the highest R^2^. Accordingly, the half-life of IgG was estimated as ~53 days and the diminish time was estimated at about 211 days post symptom onset (Fig. 1p and Supplementary Table S4).

The IgG/IgM ratio was calculated as log10(1/IgG) divided by log10(1/IgM). The dynamic change of IgG/IgM ratio was simulated as a line along the clinical course, which rose slowly till 50 days after symptom onset and then rapidly increased. A quadratic curve fitted the points with an R^2^ of 0.91, indicating a decent goodness of fit (Fig. 1q). Moreover, no factor was found to be related with the IgG/IgM ratio by ordinal logistic regression model, indicating a stable profile that weas devoid of influence from host-related factors (Supplementary Table S5).

Next, the antibody production change over the course was determined. The study period was divided into three periods: 1–28 January, 29 January–6 February, and 7 February–17 March 2020. The IgG and IgM antibody from 125, 140, and 101 COVID-19 patients were evaluated at serial time points following infection and were plotted and fitted to a linear regression. A decrease of the GMRT of the IgM and IgG was observed for the samples collected at the late period than those taken at the early epidemic and the differences of IgM and IgG were significant during 32–42 days after symptom onset among the three periods (all *P* < 0.05) (Supplementary Fig. S1 and Table S6). The effect of age, gender, days from symptom onset to admission, hypertension, diabetes, and disease severity were adjusted. The significant differences still existed after adjusting the above variables.

Altogether 267 and 363 patients were evaluated for the serum cytokines and peripheral lymphocyte subsets, respectively. Sequential evaluation disclosed a significant decrease of the evaluated lymphocyte subsets (Supplementary Fig. S2). The longitudinal profile of the serum cytokines showed an increase of IL-6 during the first week of infection (Supplementary Fig. S3). The severe patients had higher levels of IL-2R, IL-6, IL-8, and TNF-α, and lower counts of NK, B-lymphocytes, T helper cells (Th), suppressor T cell (Ts), and T lymphocytes on admission compared to the mild patients (all *P* < 0.05, Supplementary Table S7).

Finally, we performed a comprehensive correlation analysis of COVID-19-specific antibodies and all the other evaluated immunological indices, including 4 types of cytokines and 14 lymphocyte subsets. According to the spearman correlation analysis, the magnitude of IgG antibody titer was well correlated with tumor necrosis factor α (TNF-α) (*r* = 0.536), and T lymphocyte count (*r* = −0.679) (Supplementary Fig. S2). There was a negative correlation between IgM antibody titer and T lymphocyte count (*r* = −0.750). Only low correlations were observed between IgG or IgM antibody titer with the other cytokine and lymphocyte parameters (Supplementary Figs. S3 and S4).

According to our results, SARS-CoV-2-specific IgM antibody titer can reach the peak levels by the early 3–4 weeks and was predicted to last for about two months. IgG titer can also reach the peak levels at 3–4 weeks and the diminish time was estimated to be 7 months after symptom onset. Patients with older age or severe disease had higher IgM antibody level during the disease, but with delayed IgG antibody production during early infection. In contrast, patients with older age or severe disease achieved a higher IgG level than patients of younger age and mild disease. This study has revealed the magnitude and longitude of the antibody response, which could be used as a useful indicator for diagnosis and for evaluation of reinfection in case of re-exposure to the virus. Future investigation integrating the quantification of virus copy number and the titer of neutralizing antibody might further enhance our understanding of the interplay between viral replication, antibody responses, and disease progression.

## Supplementary information

Supplementary Information

## Supplementary information

**Supplementary Information** accompanies the paper at (10.1038/s41421-020-00209-2).
