# Supplementary material for: Temporal antibody responses to SARS-CoV-2 in patients of coronavirus disease 2019
Source: Cell Discov. 2020 Sep 15;6:64. doi: 10.1038/s41421-020-00209-2 (PMC7490261; doi:10.1038/s41421-020-00209-2)
Supplement: Supplementary file 1 — Supplementary Information [file 41421_2020_209_MOESM1_ESM.pdf]

Supplementary Information

Materials and methods

Patient Enrollment

The patients in the study were enrolled from the Tongji Hospital of Huazhong University of Science and Technology, which is a COVID-19 referral hospital in Wuhan, Hubei Province. All patients tested positive for SARS-CoV-2 RNA by real-time reverse transcription PCR (RT-PCR) assay and admitted to the Tongji Hospital during January–March, 2020 were eligible for participation.

Data Collection

All the epidemiologic interviews were conducted at the time of case identification to collect patient's demographics, symptom history, and relevant exposures during the 2 weeks before onset. A medical chart review was performed to collect medical history, symptoms before hospitalization and daily information regarding symptoms during hospitalization, clinical course, treatments, vital signs, diagnostic tests, SARS-CoV-2 detection results and clinical outcome. We defined symptom onset as the first day of reported symptoms consistent with COVID-19. We analyzed the data relative to the date of symptom onset (day 0).

Serologic Assays

An IgM and IgG antibody detection kit (Yahuilong Biotechnology, Shenzhen, China) was developed to detect COVID-19 envelope protein E and nucleocapsid protein N antigen during hospitalization. The background antibody titer in uninfected healthy individuals is < 10 AU/mL. Any test that is > 10 AU/mL was considered as positive.

Lymphocytes and lymphocyte subsets (T, B and NK cells) were quantified by flow cytometry.

Statistical analysis

Descriptive statistics were calculated for all variables; continuous variables were summarized as mean and standard deviation or median and interquartile range (IQR), and categorical variables were summarized as frequencies and proportions. The antibody reciprocal titers were log-transformed to allow for comparison of geometric mean reciprocal titers (GMRTs) across groups. To determine the difference between groups, independent t test, chi-square test, Fisher exact test, or non-parametric test was used where appropriate. Biologically plausible variables in the univariate analysis were entered into multivariate logistic regression model by stepwise method. IgM and IgG antibody titers were computed using non-linear generalized estimating equations (GEE) to determine the effect from considered variables on IgM and IgG antibody production and decay. Odds ratio (OR) and its 95% confidence interval (CI) were estimated using maximum likelihood method. The IgG antibody decay rate was derived from the negative slopes of the regression lines for antibody concentrations versus time in days. Half-life (t1/2) was calculated as the time required for antibody concentrations to decrease by 50% from the initial value. The differences of lymphocyte subsets were compared by the rank and general linear model between different groups. The SARS-COV-2 specific antibody titers were compared with the lymphocyte subsets and cytokine evaluated at the same time point by using the Spearman test for correlation. A two-sided P value of less than 0.05 was considered to

be statistically significant. All analyses were performed using R software (Version 3.5.1, R Foundation for Statistical Computing).

Supplementary information, Fig. S1

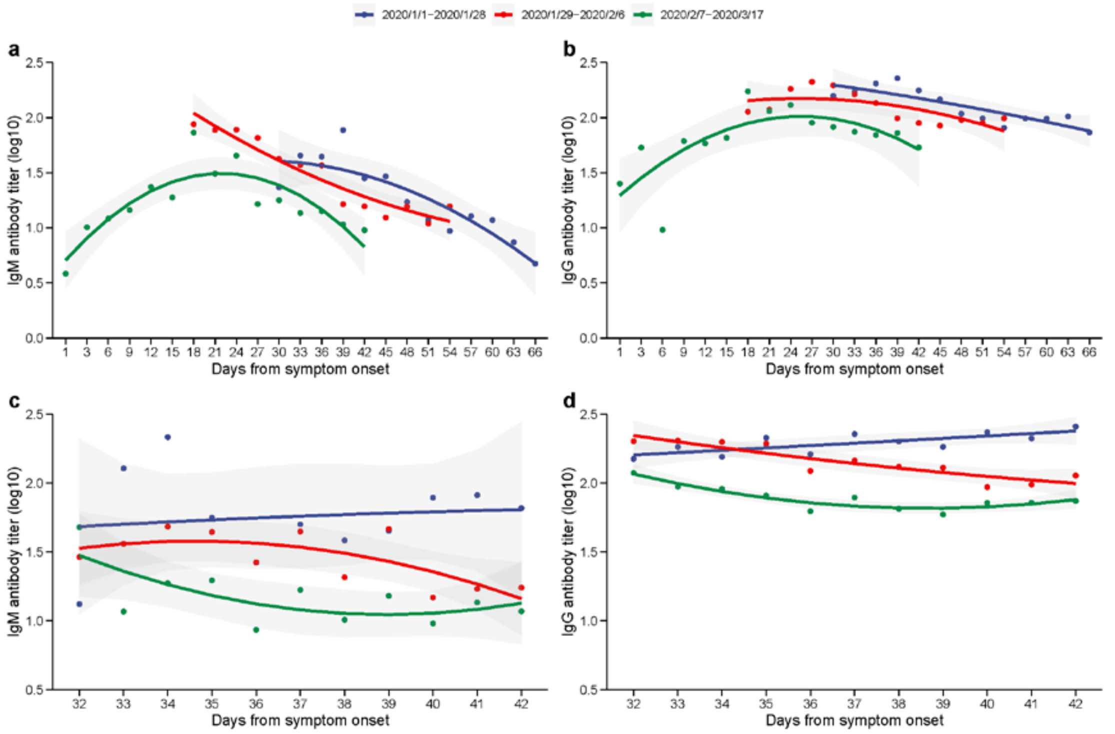

Supplementary Figure 1. Dynamic profile of the IgM and IgG level of COVID-19 patients in different hospitalization periods.

Three periods were classified based on the hospitalization of the COVID-19 patients as the following: 1st - 28th January 2020 (blue line), 29th January – 6th February 2020 (red line), and 7th February – 17th March 2020 (green line). a, IgM antibody titer in COVID-19 patients with three days interval; b, IgG antibody titer in COVID-19 patients with three days interval; c, IgM antibody titer on a daily basis during the 32-42 days period after symptom onset; d, IgG antibody titer on a daily basis during 32-42 days period after symptom onset. The comparison was performed with generalized estimating equation analysis. The measurement was performed once with the coefficient of variation value of around 5%.

Supplementary information, Fig. S2

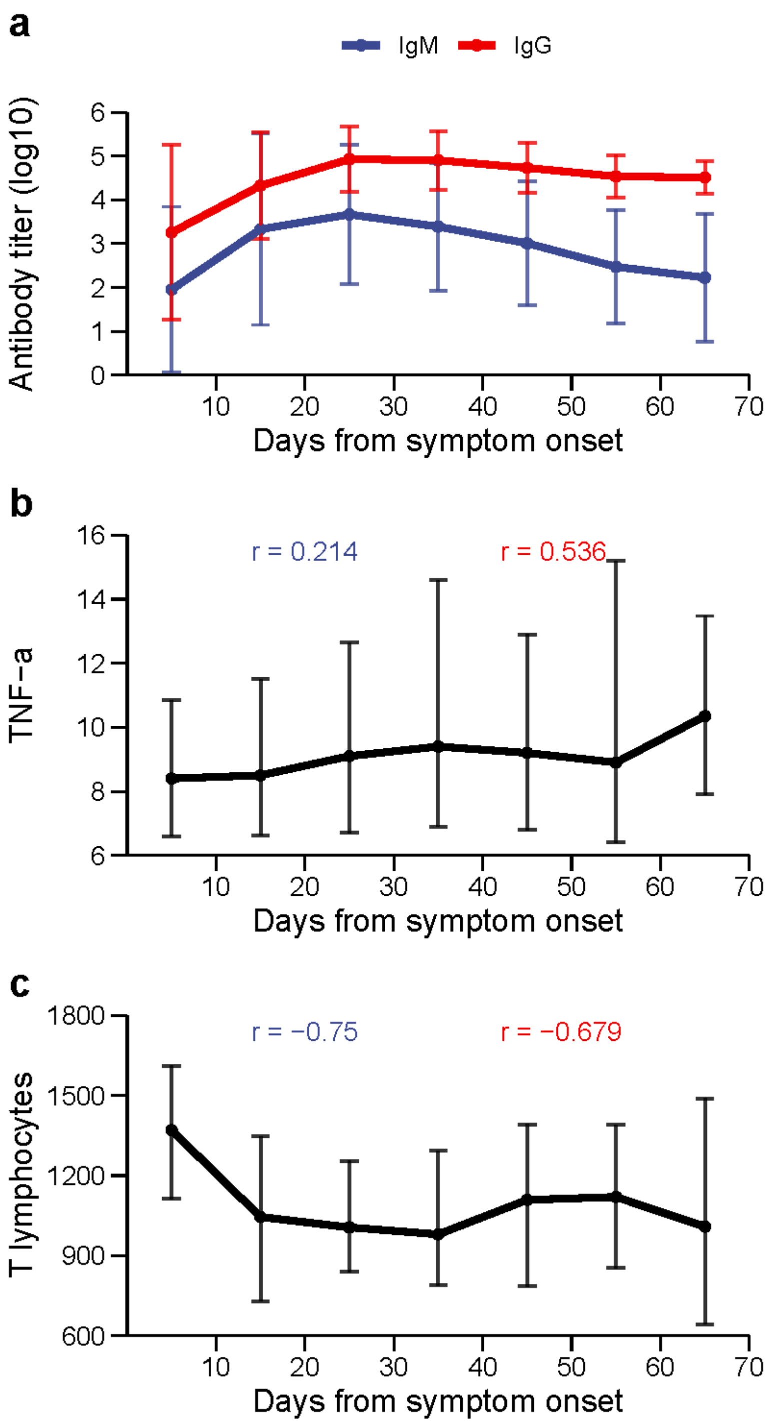

Supplementary Figure 2. Dynamic profile of the cytokine level and lymphocyte subset population showing high correlations with IgM or IgG antibody titer.

a, IgM (blue) and IgG (red) antibody titers were shown for the COVID-19 patients during the indicated time points post symptom onset; b, correlation of TNF-α with IgM (r=0.214) and IgG (r=0.536) level

during the indicated time points post symptom onset; c, correlation of T lymphocyte count with IgM (r=-0.75) and IgG (r=-0.679) level during the indicated time points post symptom onset. The median and interquartile ranges were presented for the parameters. The correlation efficient was calculated using Spearman rank correlation analysis based on any two of the curves. The r> (+/-) 0.500 was considered as a good correlation. The serum was collected for the detection of cytokines. The EDTA anti-coagulated blood samples collected for lymphocyte subsets evaluation. The measurement was performed once with the coefficient of variation value of around 5%.

Supplementary information, Fig. S3

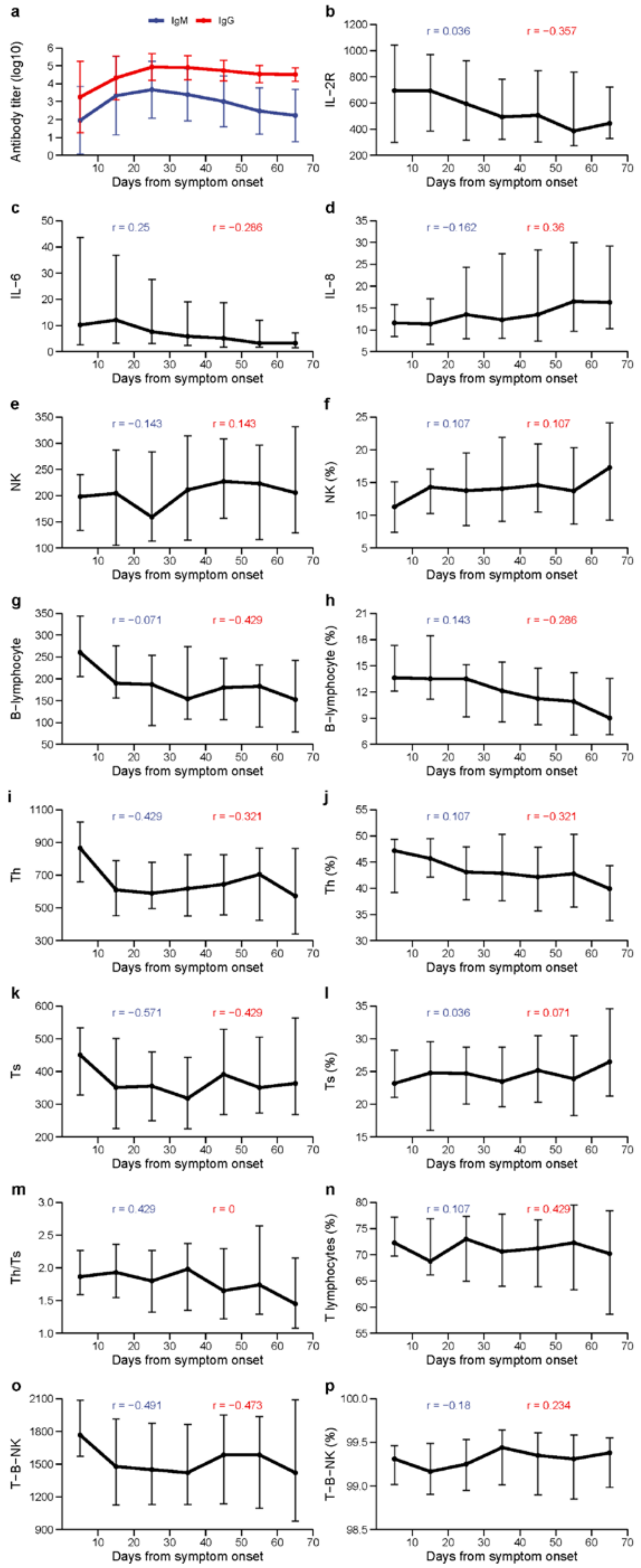

Supplementary Figure 3. Dynamic profile of the cytokine and lymphocyte subset showing low correlations with IgM or IgG antibody titer.

a, IgM (blue) and IgG (red) antibody titers were shown for the COVID-19 patients during the indicated time points post symptom onset (same data as in Sup.Fig.2a, shown here for easier comparison within the figure); b, correlation of IL-2R with IgM (r=0.036) and IgG (r=-0.357) level during the course of detection; c, correlation of IL-6 with IgM (r=0.25) and IgG (r=-0.286) level; d, correlation of IL-8 with IgM (r=-0.162) and IgG (r=0.36) level; e, correlation of NK cell counts with IgM (r=-0.143) and IgG (r=0.143) level; f, correlation of NK cell percentage with IgM (r=0.107) and IgG (r=0.107) level; g, correlation of B lymphocyte counts with IgM (r=-0.071) and IgG (r=-0.429) level; h, correlation of B lymphocyte percentage with IgM (r=0.143) and IgG (r=-0.286) level; i, correlation of Th cell counts with IgM (r=-0.429) and IgG (r=-0.321) level; j, correlation of Th cell percentage with IgM (r=0.107) and IgG (r=-0.321) level; k, correlation of Ts cell counts with IgM (r=-0.571) and IgG (r=-0.429) level; l, correlation of Ts cell percentage with IgM (r=0.036) and IgG (r=0.071) level; m, correlation of Th/Ts ratio with IgM (r=0.429) and IgG (r=0); n, correlation of T lymphocyte percentage with IgM (r=0.107) and IgG (r=0.429) level; o, correlation of total T,B,NK cell counts with IgM (r=-0.491) and IgG (r=-0.473) level; p, correlation of T-B-NK cell percentage with IgM (r=-0.18) and IgG (r=0.234) level. IL, interleukin; NK, natural killer; Th, T helper; Ts, suppressor T cell. The correlation efficient was calculated using Spearman rank correlation analysis. The measurement was performed once with the coefficient of variation value of around 5%.

Supplementary information, Fig. S4

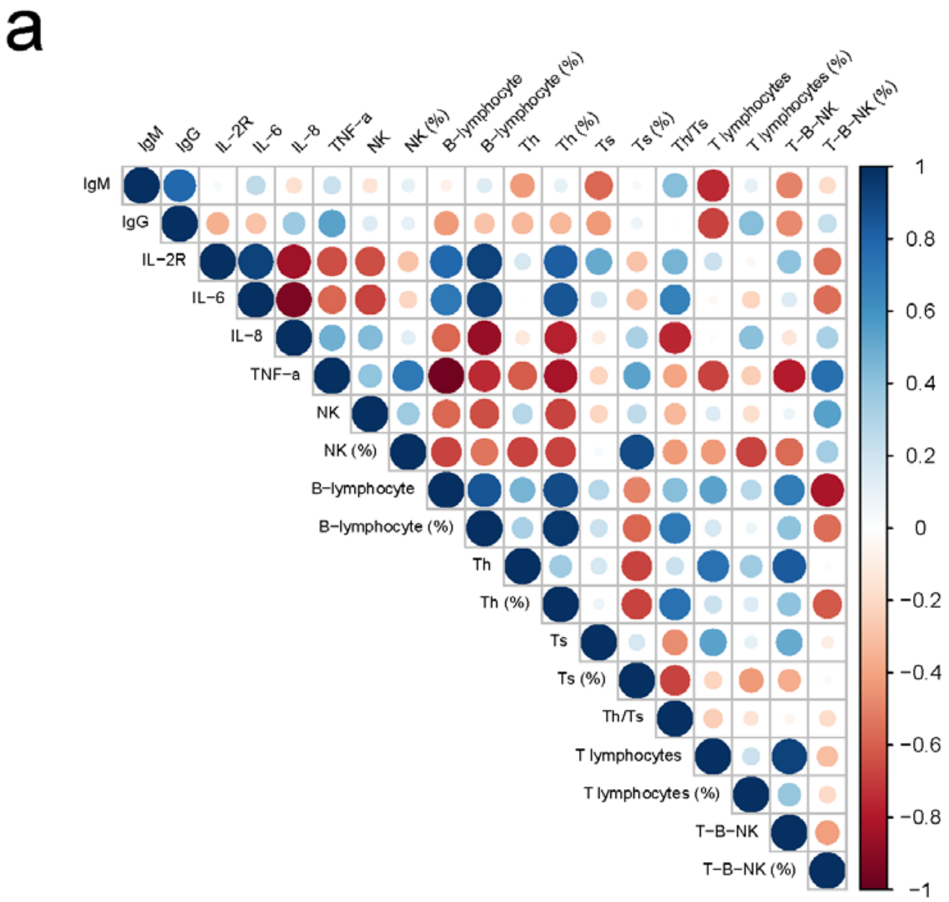

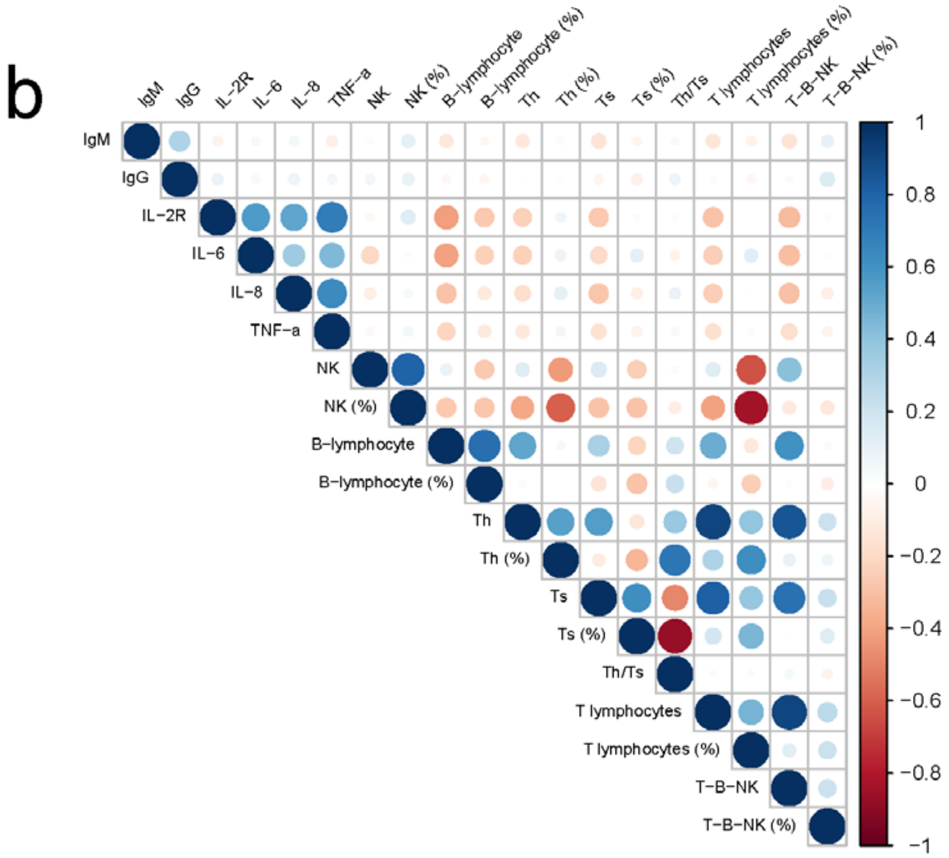

Supplementary Figure 4. Correlation matrix of IgM and IgG with cytokine and lymphocyte subsets.

The size of each dot in the triangular matrix shows the strength of correlation calculated with Spearman rank correlation analysis (positive in blue or negative in red) between the laboratory indicators. a, based on the curve data; b, based on the individual data. Strong positive correlations stand out in dark blue while strong negative correlations stand out in dark red.

Supplementary Table S1. Basic information of the COVID-19 patients in our study.

Characteristics	Total (N=366)	Mild (n=222)	Severe (n=144)	P value
Age, years, median (IQR)	62 (51-69)	60 (47-66)	66 (57-73)	<0.001*
≤60	173 (47.3)	121 (54.5)	52 (36.1)	0.001#
>60	193 (52.7)	101 (45.5)	92 (63.9)	
Sex, male, n (%)	177 (48.4)	96 (43.2)	81 (56.2)	0.015#
Days from symptom onset to admission, median (IQR)	20 (10-29)	20 (11-31)	15 (9-27)	0.001*
Length of stay, days, median (IQR)	31 (14-43)	20 (11-39)	40 (30-47)	<0.001*
Days from symptom onset to discharge/death, median (IQR)	51 (42-60)	49 (40-57)	55 (46-64)	<0.001*
Hypertension	136 (37.2)	73 (32.9)	63 (43.8)	0.036#
Diabetes	65 (17.8)	36 (16.2)	29 (20.1)	0.337#

IQR, interquartile range; COVID-19, coronavirus disease 2019. *, comparison by Kruskal-Wallis test; #, comparison by $\chi 2$ test.

Supplementary Table S2. The factors associated with IgM or IgG antibody titers of the COVID-19 patients analyzed with the generalized estimated equation.

Characteristics	IgM		IgG		IgM (1-30 days)		IgG (1-30 days)		IgM (30-70 days)		IgG (30-70 days)	
	**OR (95%CI)**	**P**	**OR (95%CI)**	**P**	**OR (95%CI)**	**P**	**OR (95%CI)**	**P**	**OR (95%CI)**	**P**	**OR (95%CI)**	**P**
**Age, years (median, IQR)**												
≤60	Reference		Reference		Reference		Reference		Reference		Reference	
>60	1.12 (1.02-1.21)	0.012	1.08 (1.03-1.13)	<0.001	1.26 (1.02-1.55)	0.035	1.11 (0.97-1.29)	0.140	1.09 (1.00-1.18)	0.049	1.06 (1.03-1.10)	0.001
**Sex, male, n (%)**												
Female	Reference		Reference		Reference		Reference		Reference		Reference	
Male	1.04 (0.96-1.13)	0.369	1.01 (0.97-1.06)	0.567	1.24 (1.00-1.54)	0.049	1.15 (1-1.34)	0.054	1.01 (0.93-1.10)	0.836	0.98 (0.95-1.01)	0.245
**Days from symptom onset to admission, median (IQR)**												
≤20	Reference		Reference		Reference		Reference		Reference		Reference	
>20	0.82 (0.75-0.89)	<0.001	0.96 (0.92-1.01)	0.097	0.79 (0.60-1.03)	0.084	1.04 (0.88-1.22)	0.668	0.80 (0.73-0.87)	<0.001	0.92 (0.88-0.95)	<0.001
**Hypertension**												
No	Reference		Reference		Reference		Reference		Reference		Reference	
Yes	0.95 (0.87-1.04)	0.256	1 (0.96-1.05)	0.869	1.09 (0.87-1.36)	0.464	0.98 (0.84-1.14)	0.777	0.91 (0.84-0.99)	0.034	1.02 (0.98-1.05)	0.401
**Diabetes**												
No	Reference		Reference		Reference		Reference		Reference		Reference	
Yes	0.99 (0.90-1.10)	0.901	1.09 (1.04-1.14)	<0.001	0.96 (0.74-1.24)	0.760	1.20 (1.02-1.39)	0.023	1 (0.90-1.10)	0.929	1.07 (1.04-1.11)	<0.001
**Disease severity**												
Mild	Reference		Reference		Reference		Reference		Reference		Reference	
Severe	1.10 (1.01-1.20)	0.032	1.05 (1-1.09)	0.038	1.27 (1.02-1.59)	0.034	1.08 (0.92-1.26)	0.370	1.09 (1-1.19)	0.059	1.03 (1-1.07)	0.074

IgM or IgG antibody titers were log_{10} transformed. OR, odds ratio; CI, confidence interval; COVID-19, coronavirus disease 2019. The generalized estimated equation was used in the analysis. IgM or IgG antibody titter was Log_{10} transformed.

Supplementary Table S3. The factors associated with the maximum value of SARS-COV-2 specific IgM and IgG antibody determined by generalized estimation equation.

Characteristics	IgM		IgG	
	OR (95%CI)	P	OR (95%CI)	P
**Age, years**				
≤60	Reference		Reference	
>60	1.13 (0.93-1.37)	0.215	1.08 (1.00-1.16)	0.047
**Sex**				
Female	Reference		Reference	
Male	0.96 (0.80-1.15)	0.656	0.95 (0.89-1.02)	0.158
**Days from symptom onset to admission**				
≤20	Reference		Reference	
>20	0.78 (0.64-0.95)	0.014	0.88 (0.82-0.94)	<0.001
**Hypertension**				
No	Reference		Reference	
Yes	0.87 (0.72-1.06)	0.158	0.98 (0.91-1.05)	0.583
**Diabetes**				
No	Reference		Reference	
Yes	0.95 (0.76-1.19)	0.659	1.08 (0.99-1.18)	0.071
**Disease severity**				
Mild	Reference		Reference	
Severe	1.10 (0.91-1.32)	0.329	0.99 (0.93-1.06)	0.803

The maximum value of IgM determined during 10-40 days after symptom onset and the maximum value of IgG determined during 20-70 days after symptom onset were used. The IgM or IgG antibody titers were Log_{10} transformed.

Supplementary Table S4. Dynamic profile on antibody titers of IgM and IgG fitted at different time intervals of COVID-19 patients by quadratic curves.

Group	Predictor formula	R^2	Days of maximum value	Days of converting negative
**IgM**				
2-day	$y=2.08-0.0134x-0.0000827x^2$	0.91	21	59
3-day	$y=2.06-0.0153x-0.0000573x^2$	0.92	18	57
5-day	$y=1.9822-0.01x-0.0001x^2$	0.97	20	61
10-day	$y=1.9733-0.0139x-0.00003x^2$	0.99	25	62
**IgG**				
2-day	$y=2.26-0.00345x-0.0000222x^2$	0.58	21	173
3-day	$y=2.24-0.00304x-0.0000276x^2$	0.75	18	164
5-day	$y=2.31-0.00552x-0.00000464x^2$	0.92	25	203
10-day	$y=2.27-0.00464x-0.00000648x^2$	0.93	25	211

Supplementary Table S5. The factors associated with IgG/IgM ratio of COVID-19 patients by ordinal logistic regression model.

Characteristics	Low group *vs.* moderate group		High group *vs.* moderate group	
	**OR (95% CI)**	**P**	**OR (95% CI)**	**P**
**Age, years**				
≤60				
>60	0.7 (0.2-2.53)	0.591	1.12 (0.49-2.57)	0.79
**Sex, male**				
Female				
Male	1.45 (0.44-4.77)	0.536	0.78 (0.37-1.67)	0.525
**Days from symptom onset to admission**				
≤20				
>20	2.17 (0.64-7.3)	0.212	1.15 (0.53-2.51)	0.720
**Hypertension**				
No				
Yes	1.09 (0.29-4.08)	0.901	1.44 (0.64-3.25)	0.373
**Diabetes**				
No				
Yes	1.78 (0.44-7.13)	0.416	0.88 (0.32-2.43)	0.808

The ordinal logistic regression model was used. Median group represents the value of IgG/IgM ratio was within the range of mean±SD at each time point. Low group represents the value of IgG/IgM ratio was lower than the value of mean-SD at each time point. High group represents the value of IgG/IgM ratio was higher than the value of mean+SD at each time point. SD, standard deviation. OR, odds ratio. CI, confidence interval. COVID-19, coronavirus disease 2019. IgG/IgM ratio was calculated as Log_{10}(1/IgG) divided by Log_{10}(1/IgM).

Supplementary Table S6. The comparison of IgM and IgG antibody titer at the three periods during 32-42 days after symptom onset by logistic regression model.

Characteristics	Crude OR (95% CI)	P	Adjusted OR (95% CI)*	P
IgM				
2020/1/1−2020/1/28	Reference		Reference	
2020/1/29−2020/2/6	0.76 (0.64-0.91)	0.002	0.77 (0.65-0.91)	0.002
2020/2/7−2020/3/17	0.56 (0.46-0.68)	<0.001	0.58 (0.48-0.7)	<0.001
IgG				
2020/1/1−2020/1/28	Reference		Reference	
2020/1/29−2020/2/6	0.86 (0.82-0.91)	<0.001	0.86 (0.81-0.9)	<0.001
2020/2/7−2020/3/17	0.67 (0.63-0.71)	<0.001	0.67 (0.63-0.71)	<0.001

*The logistic regression model was adjusted for age, sex, days from symptom onset to admission, hypertension, diabetes and disease severity. OR, odds ratio; CI, confidence interval.

Supplementary Table S7. The cytokine and lymphocyte subsets in the COVID-19 patients on admission

Laboratory parameters	Total (N=366)	Mild (n=222)	Severe (n=144)	P value
**IL-2R**	458 (285-848)	358 (259-632)	794 (476-1077)	<0.001
**IL-6**	4.5 (2.0-18.2)	2.9 (1.6-7.2)	18.5 (6.0-42.1)	<0.001
**IL-8**	11.3 (7.3-17.5)	10.1 (6.6-14.9)	13.5 (9.1-21.1)	<0.001
**TNF-α**	8.0 (6.4-10.8)	7.7 (6.1-9.9)	9.2 (7.2-11.9)	<0.001
**NK count**	220 (134-312)	232 (160-328)	174 (109-271)	<0.001
**NK (%)**	14.5 (9.5-21.2)	15.0 (10.1-21.4)	13.9 (8.8-20.9)	0.289
**B-lymphocyte count**	180 (107-260)	202 (135-277)	144 (72-224)	<0.001
**B-lymphocyte (%)**	11.8 (8.5-15.2)	12.0 (9.1-15.1)	11.4 (7.1-15.9)	0.243
**Th count**	640 (474-826)	710 (527-872)	548 (385-711)	<0.001
**Th (%)**	42.0 (37.0-48.1)	42.4 (37.6-48.5)	41.5 (35.5-47.2)	0.168
**Ts count**	370 (261-509)	406 (282-523)	334 (213-482)	0.003
**Ts (%)**	24.6 (19.9-30.1)	24.3 (19.8-29.2)	24.9 (20.2-31.3)	0.307
**Th/Ts**	1.8 (1.3-2.3)	1.8 (1.4-2.3)	1.7 (1.2-2.6)	0.337
**T lymphocytes count**	1087 (825-1372)	1156 (919-1446)	976 (648-1176)	<0.001
**T lymphocytes (%)**	71.1 (64.7-77.3)	71.1 (65.2-77.3)	71.1 (62.8-77.6)	0.923
**T-B-NK count**	1527 (1152-1932)	1672 (1356-2040)	1338 (999-1752)	<0.001
**T-B-NK (%)**	99.3 (99.0-99.6)	99.3 (99.0-99.6)	99.4 (98.8-99.6)	0.560

IL, interleukin. TNF, tumor necrosis factor; COVID-19, coronavirus disease 2019; NK, natural killer cells; Th, T helper cells; Ts, suppressor T cell. The comparison between mild and severe patients was performed by Kruskal-Wallis test.
